# Functional community structure of African monodominant *Gilbertiodendron dewevrei* forest influenced by local environmental filtering

**DOI:** 10.1002/ece3.2589

**Published:** 2016-12-20

**Authors:** Elizabeth Kearsley, Hans Verbeeck, Koen Hufkens, Frederik Van de Perre, Sebastian Doetterl, Geert Baert, Hans Beeckman, Pascal Boeckx, Dries Huygens

**Affiliations:** ^1^ Department of Applied Ecology and Environmental BiologyComputational and Applied Vegetation Ecology – CAVElabGhent UniversityGentBelgium; ^2^ Department of Applied Analytical and Physical ChemistryIsotope Bioscience Laboratory – ISOFYSGhent UniversityGentBelgium; ^3^ Service of Wood BiologyRoyal Museum for Central AfricaTervurenBelgium; ^4^ Department of Organismic and Evolutionary BiologyHarvard UniversityCambridgeMAUSA; ^5^ Evolutionary Ecology GroupUniversity of AntwerpAntwerpBelgium; ^6^ Institute of GeographyAugsburg UniversityAugsburgGermany; ^7^ Department of Applied BiosciencesGhent UniversityGentBelgium; ^8^ Instituto Multidisciplinario de Biología VegetalUniversidad Nacional de Córdoba & CONICETCordobaArgentina

**Keywords:** Democratic Republic of Congo, environmental filtering, functional traits, *Gilbertiodendron dewevrei*, plant population and community dynamics, single‐species dominance, species establishment

## Abstract

Monodominant patches of forest dominated by *Gilbertiodendron dewevrei* are commonly found in central African tropical forests, alongside forests with high species diversity. Although these forests are generally found sparsely distributed along rivers, their occurrence is not thought to be (clearly) driven by edaphic conditions but rather by trait combinations of *G. dewevrei* that aid in achieving monodominance. Functional community structure between these monodominant and mixed forests has, however, not yet been compared. Additionally, little is known about nondominant species in the monodominant forest community. These two topics are addressed in this study. We investigate the functional community structure of 10 one‐hectare plots of monodominant and mixed forests in a central region of the Congo basin, in DR Congo. Thirteen leaf and wood traits are measured, covering 95% (basal area weighted) of all species present in the plots, including leaf nutrient contents, leaf isotopic compositions, specific leaf area, wood density, and vessel anatomy. The trait‐based assessment of *G. dewevrei* shows an ensemble of traits related to water use and transport that could be favorable for its location near forest rivers. Moreover, indications have been found for N and P limitations in the monodominant forest, possibly related to ectomycorrhizal associations formed with *G. dewevrei*. Reduced leaf N and P contents are found at the community level for the monodominant forest and for different nondominant groups, as compared to those in the mixed forest. In summary, this work shows that environmental filtering does prevail in the monodominant *G. dewevrei* forest, leading to lower functional diversity in this forest type, with the dominant species showing beneficial traits related to its common riverine locations and with reduced soil N and P availability found in this environment, both coregulating the tree community assembly.

## 1 Introduction

Tropical rain forests are complex systems with high diversity of tree species growing in three continents along the equator. However, large‐scale inventory networks across the tropics have shown that there are important intercontinental differences in rain forest tree communities. African tropical rain forests, for example, are less diverse in terms of tree species than Amazonian and South‐East Asian rain forests (Parmentier et al., 2007; Slik et al., 2015). In all these regions, however, lower diversity forests are found in the form of monodominant forest, where a single canopy species constitutes ≥60% of all canopy‐level trees (Connell & Lowman, 1989; Hart, 1985; Peh, Lewis, & Lloyd, 2011). Such monodominance in old‐growth forests can be caused by distinct edaphic conditions (Richards, 1996), for example, in water‐logged forest (Connell & Lowman, 1989; Richards, 1996) and low‐nutrient forests (McGuire, 2007). Another type of monodominance is related to the disturbance regime of the forest, with low disturbance rates favoring competitive exclusion (Connell & Lowman, 1989; Hart, Hart, & Murphy, 1989). Yet, monodominant forests can also present themselves in similar environmental conditions as their adjacent high‐diversity forest and are apparently not bound to major edaphic differences or recent disturbances. Research on mechanisms to explain monodominance has focused on traits of dominant species that provide a competitive advantage, including low leaf litter decomposition rates, high seedling shade tolerance, large seed size, and defense against herbivory (Hart et al., 1989; Torti, Coley, & Kursar, 2001). The best‐studied of these monodominant forests in Africa is that dominated by *Gilbertiodendron dewevrei* (De Wild.) J. Leonard from which naturally occurring monodominant patches are commonly found across central Africa, alongside forests with high species diversity (Fayolle et al., 2014; Hart, 1985; Peh, Sonké, Lloyd, Quesada, & Lewis, 2011; Viennois, Barbier, Fabre, & Couteron, 2013). Some environmental differences between these monodominant forests and adjacent mixed forests have been described, although the presence of an environmental filter (i.e., ecological filters related to the abiotic environment selecting for species suitable for this environment; Keddy, 1992) for establishment of the monodominance is not always evident. For example, these forests are often found along rivers (Fayolle et al., 2014), although not exclusively (Hart et al., 1989). Additionally, Peh, Sonké et al. (2011) did not find evidence for differences in soil characteristics between this monodominant forest and the adjacent mixed forest, similar to findings of Hart (1985) and Conway (1992), while Torti et al. (2001) did report lower availability of nitrogen in the monodominant forest. Moreover, how the presence of this monodominance and its according environmental conditions impacts the overall tree community, species composition, and richness is not well understood. Variable species diversity of monodominant forests compared to the adjacent mixed forest has been reported, with both similar (Connell & Lowman, 1989; Makana, Terese, Hibbs, & Condit, 2004) and lower species diversity (Djuikouo, Sonké, Doucet, Nguembou, & Lewis, 2010; Hart et al., 1989; Peh, 2009). Nevertheless, low species diversity does not necessarily indicate an equivalently low functional diversity in the community. Functional diversity, defined as the value, range, and distribution of functional traits in a given ecosystem (Díaz et al., 2007), namely also depends on variability of trait values of all species present, both within and between species, and on the extent of overlap of functional niches. Moreover, this distribution of trait values of all individuals in a community depends on the balance between processes related to environmental filtering and those leading to niche differentiation between individuals (Harper, 1977; MacArthur & Levins, 1967).

Within this study, we investigate functional diversity and functional community structure in 10 one‐hectare plots of mixed (*n* = 5) and monodominant *Gilbertiodendron dewevrei* (*n* = 5) forests in a central region of the Congo Basin, in DR Congo. A dataset of 13 leaf and wood traits was used focusing on traits with a clear link to resource acquisition, growth, nutrient cycling, and decomposition, covering 95% (basal area weighted) of all species present in the plots. Plant traits are scaled up from individuals to community‐level trait distributions as a means to investigate ecosystem functioning and community assembly through environmental filtering (e.g., Fayolle et al., 2012; Fortunel, Paine, Fine, Kraft, & Baraloto, 2014; Laughlin, Fulé, Huffman, Crouse, & Laliberté, 2011). We hypothesize (Hypothesis I) that *G. dewevrei* monodominant forests hold a lower functional diversity than the mixed forest related to the existence of environmental filtering. Even though the dominance of *G. dewevrei* could not be linked to edaphic controls in previous studies, recent studies indicate that (1) *G. dewevrei* monodominant forests most commonly occur along rivers and forest streams (Fayolle et al., 2014) and (2) dominant species can modify soil conditions (Brookshire & Thomas, 2013) which in turn can act as an environmental filter. Furthermore, we hypothesize (Hypothesis II) that other species present in this monodominant forest contain (an ensemble of) traits similar to those of *G. dewevrei* as they encounter the same environmental filtering and that species that do not possess these traits will be confined to the mixed forest.

## 2 Materials and Methods

### 2.1 Study area

This study was carried out in the UNESCO Biosphere reserve in Yangambi, ±100 km west of Kisangani, DR Congo. The reserve covers an area of 6,297 km^2^ just north of the Congo River, and all study sites are located in the southwestern part of the reserve (N00°48′; E24°29′). As measured in the Yangambi reserve, the region receives an annual precipitation of 1839.5 ± 205.7 mm (1980–2012) with an average dry season length of 3.3 ± 1.3 months with monthly precipitation lower than 100 mm, during December–February. Temperatures are high and constant throughout the year with a minimum of 24.2 ± 0.4°C in July and a maximum of 25.5 ± 0.6°C in March. Soils in the Yangambi plateau are Xanthic Ferralsols (WRB 2014), primarily formed from fluvio‐eolian sediments, composed mostly of quartz sand, kaolinite clay, and hydrated iron oxides (Van Ranst, Baert, Ngongo, & Mafuka, 2010).

Permanent sampling plots of one hectare were installed and measured in 2012 (Kearsley et al., 2013) in old‐growth mixed forest (*n* = 5) and old‐growth monodominant forest (*n* = 5) dominated by *Gilbertiodendron dewevrei* (De Wild.) J. Leonard (Table 1). Monodominant forest was found near forest streams, while mixed forest was found covering the entire reserve. Permanent sampling plots of the mixed forest plots were located within a radius of 2 km of the monodominant forest plots. The permanent plot setup in Yangambi does not contain mixed forest plots near forest streams. Within all plots, all trees with a DBH ≥ 10 cm have been measured for DBH and identified to species level. For each taxon, at least one herbarium specimen and one silica gel dried leaf sample were collected. Vouchers were deposited in the Herbarium Yangambi (DRC) and in the herbarium of the Botanic Garden Meise (Belgium). In order to verify field identifications, vouchers were compared with reference specimens kept by the Botanic Garden Meise for both morphologic and genetic characteristics (barcodes generated using rbcL and matK sequences as recommended by the CBOL Plant Working Group 2009).

**Table 1:** Stand characteristics and mean estimated diversity indices (expressed in effective number of species) for the mixed and monodominant forests. Species abundance is basal area weighted, with only the five most abundant species shown (full species names: *Scorodophloeus zenkeri, Petersianthus macrocarpus, Panda oleosa, Anonidium mannii, Tridesmostemon omphalocarpoides, Gilbertiodendron dewevrei, Cavacoa quintasii, Cleistanthus mildbraedii,* and *Dialium pachyphyllum*). Letters indicate whether there is a significant difference (*p* < .01) between the forest types

	Mixed forest	Monodominant forest
Stand characteristics		
Stem density (per ha)	412 ± 85 (a)	343 ± 80 (a)
Basal area (m^2^/ha)	31.8 ± 4.1 (a)	29.7 ± 2.6 (a)
Species diversity		
Species (5 most abundant, %)	*S. zenkeri* (16.7%)	*G. dewevrei* (65.3%)
	*P. macrocarpus* (7.0%)	*C. quintasii* (6.0%)
	*P. oleosa* (5.8%)	*S. zenkeri* (5.5%)
	*A. mannii* (5.0%)	*C. mildbraedii* (2.5%)
	*T. omphalocarpoides* (4.9%)	*D. pachyphyllum* (2.0%)
Species richness	67.2 ± 6.2 (a)	46.4 ± 6.8 (b)
Pielou's evenness	0.84 ± 0.03 (a)	0.72 ± 0.03 (b)
Shannon diversity	34.4 ± 4.7 (a)	16.2 ± 2.6 (b)
Simpson diversity	21.2 ± 3.6 (a)	8.4 ± 1.2 (b)
Functional diversity		
Functional evenness	0.866 ± 0.007 (a)	0.871 ± 0.009 (a)
Functional richness	2755 ± 764 (a)	1282 ± 450 (b)
Functional divergence	0.80 ± 0.01 (a)	0.81 ± 0.01 (a)

### 2.2 Soil sampling and analysis

Within each plot, 10 soil cores have been taken using a soil auger (a standard one‐piece Dutch auger, 7 cm diameter) in three depth increments: 0–30, 30–60, and 60–90 cm. These 10 sampling locations were spatially well distributed following an “S” curve across each 1‐ha plot. All samples were oven‐dried (50°C), and the following parameters have been measured on plot‐level composite soil samples per depth increment: bulk density, carbon and nitrogen content, isotopic composition of carbon (δ^13^C) and nitrogen (δ^15^N), and bioavailable phosphorus. For two plots per forest type, more detailed soil measurements have been made, namely soil texture, pH_CaCl2_, potential cation exchange capacity (CEC_pot_), and base saturation. C and N content and isotopic compositions were analyzed using an elemental analyzer (ANCA‐SL, SerCon, Crewe, UK) coupled to an isotope ratio mass spectrometer (20‐20, SerCon, Crewe, UK) (EA‐IRMS). Bulk density was determined on composites of 10 samples per plot using Kopecky cylinders. Soil texture was determined by means of the percentage of sand, silt, and clay. All analyses were performed on air‐dried fine soil fractions (<2 mm). The sand fraction (>63 μm) was separated by wet sieving; the silt and clay fractions were determined by the Köhn pipette method after dispersion with sodium hexametaphosphate (Pansu & Gautheyrou, 2006). Soil pH_CaCl2_ was determined potentiometrically in 25 ml 0.01 M CaCl_2_ (1:2.5 soil:solution ratio) with a glass electrode using a portable multiparameter Meter HI9828 (Hanna Instruments US Inc., USA). CEC_pot_ was determined by quantifying ${\text{NH}}_{4}^{+}$ exchanged with 2 M KCl after saturating cation exchange sites with ammonium acetate buffered at pH 7.0 and measured with ICP‐MS. Exchangeable Al was extracted by 1 M KCl solution and determined colorimetrically. Resin‐extractable P was determined using resin‐impregnated membrane strips (Sharpley, 2009).

### 2.3 Trait collection and analysis

Leaf samples and wood samples from the stem were collected for all species covering a cumulative 95% basal area of each plot; that is, species were ranked from highest to lowest basal area with species included in the selection until the cutoff of 95% basal area was reached. If multiples of the same species were present in a plot, two individuals were selected for sample collection within each preassigned diameter class of 10–20, 20–30, 30–50, and >50 cm DBH. A total of 728 individuals were sampled, covering 104 species, 67 genera, 29 families. *Scorodophloeus zenkeri* Harms, *Gilbertiodendron dewevrei* (De Wild.) J. Léonard, *Garcinia punctata* Oliv., *Dialium pachyphyllum* Harms, and *Carapa procera* DC. are some of the most abundant species in the collected dataset. All samples were collected between March and May 2012.

From each individual tree, 10 leaves were sampled at various tree heights covering the range of the tree crown (i.e., both sun and shade leaves), which were fully expanded and showed no signs of pathogens or herbivory. Fresh weight of the leaf samples was measured as a composite sample, and high‐resolution images were taken to determine leaf area, while leaves were flattened between transparent Plexiglas. Leaf surface is determined by analyzing these images using ImageJ software (from the US National Institutes of Health; https://imagej.nih.gov/ij/). Leaves were subsequently dried at 60°C for 72 h, or until no more weight change occurred, and dry mass was determined. Leaf dry matter content (LDMC, leaf dry weight divided by fresh weight) and specific leaf area (SLA, leaf area divided by dry weight) were determined. Next, chemical analysis of the leaves was performed at the Isotope Bioscience Laboratory (Ghent University, Belgium). Composite leaf samples were ground to fine powder using a ball mill (ZM200; Retsch, Germany). Mass‐based leaf carbon content (LCC) and leaf nitrogen content (LNC) and the isotopic composition of carbon (δ^13^C) and nitrogen (δ^15^N) were determined using an elemental analyzer (ANCA‐SL; SerCon, Crewe, UK) coupled to an isotope ratio mass spectrometer (20‐20; SerCon, Crewe, UK) (EA‐IRMS). Isotope ratios were expressed in delta notation relative to Vienna Pee Dee Belemnite (VPDB) standard for δ^13^C and atmospheric air for δ^15^N. Leaf phosphorus content (LPC) and isotopic composition of oxygen (δ^18^O) were determined on a subset of samples, with priority to species with more replicates. Samples were retained if more than three individuals from a species were measured within a forest type, of which three to five individuals were randomly selected for analysis. In total, 358 individuals covering 76 species were measured for LPC and δ^18^O. For LPC determination, samples were prepared using the Chapman & Pratt (1961) procedure with some slight modifications and measured using the auto analyzer method, No.G‐103‐93 Rev.2 (Multitest MT7/MT8). δ^18^O is analyzed using a high‐temperature furnace interfaced with an EA‐IRMS (20‐20, SerCon, Crewe, UK). δ^18^O is expressed relative to the Vienna Standard Mean Ocean Water (VSMOW2) standard.

Wood samples with an average size of 5 × 5 × 5 cm^3^ are taken under the bark for all species with at least three replicates. The volume of the fresh sample was taken using the water displacement method. Samples were subsequently dried in an oven at 60°C until completely dry, and dry weight was measured. Wood density (WD) could then be determined as the ratio of oven dry weight divided and fresh volume. Wood vascular traits are measured on already prepared sections of slides from the xylarium of the Royal Museum of Central Africa (Tervuren, Belgium). Species were selected which match the species sampled at the inventory sites, amounting to 62 species, and three slides were used for measurements. Vessel diameters (VDm) were measured on a minimum of 30 vessels in both horizontal and vertical directions, and an average VDm is determined for each sample. All vessels were counted within a known area to determine vessel density (VD).

### 2.4 Species and functional diversity

A comparison is made of the tree species diversity in the mixed and monodominant forests, represented by diversity indices: species richness, Pielou's evenness (Pielou, 1969), Shannon–Weaver (Shannon & Weaver, 1949) and Simpson (Simpson, 1949) indices. Each metric is expressed in effective number of species in order to estimate “true” biodiversity (Jost, 2006). Effective numbers of species derived from standard diversity indices share a common set of intuitive mathematical properties and behave as one would expect of a diversity index, while raw indices do not (Jost, 2006). As species richness depends on the number of individuals sampled, irrespective of plot size, samples were standardized for their completeness. The sample completeness is the proportion of the total number of individuals in a community that belong to the species represented in the sample and can be estimated based on the sampling curves (Chao & Jost, 2012). Indices and sample completeness are calculated on a plot level (1 ha) using the functions provided in the package iNEXT (Hsieh, Ma, & Chao, 2013) in R 2.13.1 (CRAN core development team).

Multivariate statistical analysis is performed for characterizing the functional diversity because the ecology of species inherently relates to a combination of traits. Multivariate functional diversity indices are calculated for each plot. Functional diversity consists of different dimensions, and according to Mason, Mouillot, Lee, & Wilson (2005) and Villéger, Mason, & Mouillot (2008), at least three different indices are needed to capture these, namely functional richness, functional evenness, and functional divergence. Functional richness is defined as the amount of niche space filled by species in the community, thus describing trait dissimilarity. Functional richness is measured for each plot as the convex hull volume encompassing all traits. Functional evenness is the evenness of abundance distribution in filled niche space. Functional divergence is the degree to which abundance distribution in niche space maximizes divergence in functional characters within the community. Functional divergence is calculated relative to the centroid per plot. All indices are calculated using the R package FD (Laliberté, Legendre, & Shipley, 2014).

### 2.5 Functional community structure

Plot‐level community‐weighted means (CWM) of all individual traits are investigated. Intraspecific trait variability is accounted for by setting up species‐specific uniform distributions between the minimum and maximum values measured for each species. Next, a random value from within this distribution is assigned to all individuals from the same species that have not been measured. We acknowledge that the uniform distribution is not optimal to represent intraspecific trait variability, although we believe this representation better reflects the community trait assembly than using a species‐specific mean. Accordingly, with all individual trees being assigned a trait value, species abundance is taken into account for the calculation of CWM. Note that the CWM for the monodominant forest is highly influenced by the dominant species *G. dewevrei* representing 24.2% of all individuals in this community and 65.3% of the basal area.

To investigate the effect of monodominance on the community trait composition, the functional characteristics of more detailed species groups are investigated. Three species groups are defined based on species absence/presence in the two forest types, namely group 1: tree species uniquely found in the monodominant forest; group 2: species uniquely found in the mixed forest; and group 3: species found in both the mixed and monodominant forests. These species groups are indicated throughout the text as “unique species” or “shared species” for the respective forest types. This species distinction between the mixed and monodominant forest is tested using detrended correspondence analysis weighted using species basal areas (Figure S1). The analysis of trait composition of each group in each forest type accounts for the amount of individual trees of each species present, similar to the calculation of CWM, and therefore also accounts for intraspecific trait variability. Species group 3 addressing shared species in the two forest types will differ in trait composition through difference in species abundance in the two forest types.

## 3 Results

### 3.1 Stand characteristics and soil properties

The monodominant and mixed forest plots show similar basic stand characteristics with similar stem density and basal area (Table 1). Moreover, overall soil properties are similar (Table 2). Soil texture is predominantly sand (__75% sand). Bulk density is similar with an overall average of 1.4 ± 0.2 g/cm^3^ and 1.5 ± 0.1 g/cm^3^ for monodominant and mixed forest, respectively. Typical for these types of tropical soils, pH_CaCl2_ values were highly acidic (3.7–4.6) accompanied with very low CEC_pot_ values (3.1–6.3 cmol(+) per kg). Exchangeable Al ranges are highly variable between and within the two forest types, with values ranging between 15 and 146 mg/kg, decreasing with soil depth. The concentrations of exchangeable cations were similar between both forest types. N contents were similar between the two forest types and decreased with soil depth. Bioavailable P was marginally significantly higher (*p* < .05) in mixed forest (8.6 ± 1.8 mg/kg) compared to monodominant forest (6.3 ± 1.1 mg/kg) for the 0–30 cm depth increment, similar at the other soil depths.

**Table 2:** Physical and chemical soil properties for both forest types from mixed samples for each investigated depth layer. Raw data are provided for soil parameters only measured in two plots per forest type (base cations, CEC_pot_ (potential cation exchange capacity), Ex. Al (exchangeable Al), pH and texture). Mean and standard deviations are provided for N, δ^15^N, C, δ^13^C, Bio‐P (bioavailable P), and BD (bulk density). Letters indicate whether a significant difference (*p* < .01) is found for these parameters between the forest types at specific soil depths (small letter for 0–30 cm; capital letter for 30–60 cm and dash (/) small letter for 60–90 cm)

Soil depth (cm)	Mixed			Monodominant		
	0–30	30–60	60–90	0–30	30–60	60–90
Ca (mg/kg)	128–135	122–130	124–126	120–136	120–128	123–131
K (mg/kg)	33.3–35.1	13.6–22.6	8.3–21.4	16.6–62.9	10.6–17.2	10.2–15.1
Mg (mg/kg)	15.3–16.5	8.6–10.3	7.2–7.7	7.7–11.4	6.1–8.4	5.7–7.9
Na (mg/kg)	7.5–7.9	7.2–7.9	7.2–8.3	7.9–10.6	7.3–8.3	7.8–8.2
CEC_pot_ (cmol(+) per kg)	3.8–6.7	3.1–4.9	3.8–4.3	3.2–6.3	3.1–6.2	3.2–3.4
Ex. Al (mg/kg)	15.3–100	24.7–65.4	17.1–62.8	94.7–146	50.4–69.1	25.3–35.3
pH	3.7–4.3	4.0–4.4	4.2–4.5	3.9–4.1	4.2–4.3	4.4–4.6
Sand (%)	83.6–86.6	81.5–85.5	77.9–83.0	80.4–88.4	76.9–89.8	74.8–87.8
Silt (%)	1.9–1.9	2.2–3.0	1.7–1.8	3.0–3.1	2.8–3.4	2.4–3.6
Clay (%)	11.6–14.6	11.4–16.3	15.2–20.4	8.5–16.6	6.7–20.3	9.8–21.6
Bio‐P (mg/kg)	8.6 ± 1.8 (a)	4.5 ± 2.5 (A)	2.0 ± 2.0 (/a)	6.3 ± 1.1 (a)	2.1 ± 0.5 (A)	1.4 ± 0.2 (/a)
N (%)	0.10 ± 0.04 (a)	0.05 ± 0.00 (A)	0.03 ± 0.01 (/a)	0.12 ± 0.05 (a)	0.05 ± 0.01 (A)	0.03 ± 0.01 (/a)
δ^15^N (‰)	8.6 ± 1.3 (a)	9.8 ± 1.4 (A)	8.7 ± 1.5 (/a)	8.2 ± 1.4 (a)	9.8 ± 1.3 (A)	9.8 ± 1.2 (/a)
C (%)	1.30 ± 0.11 (a)	0.59 ± 0.02 (A)	0.42 ± 0.02 (/a)	1.84 ± 0.11 (b)	0.84 ± 0.02 (B)	0.54 ± 0.03 (/b)
δ^13^C (‰)	−28.3 ± 0.5 (a)	−27.2 ± 0.3 (A)	−26.5 ± 0.3 (/a)	−28.3 ± 0.5 (a)	−26.9 ± 0.7 (A)	−26.1 ± 0.4 (/a)
BD (g/cm^3^)	1.4 ± 0.2 (a)	1.5 ± 0.2 (A)	1.5 ± 0.2 (/a)	1.2 ± 0.2 (a)	1.5 ± 0.1 (A)	1.4 ± 0.1 (/a)

### 3.2 Species indices

Lower species diversity is found in the monodominant forest compared to the mixed forest, with a lower overall and rarified species richness, species evenness, and Simpson's diversity (Table 1, Figure S2). The monodominant forest has a significantly lower (*p* < .01) functional richness compared to the mixed forest. Functional evenness and divergence are similar for both forest types.

### 3.3 Monodominant forest functional community

**Figure 1:**
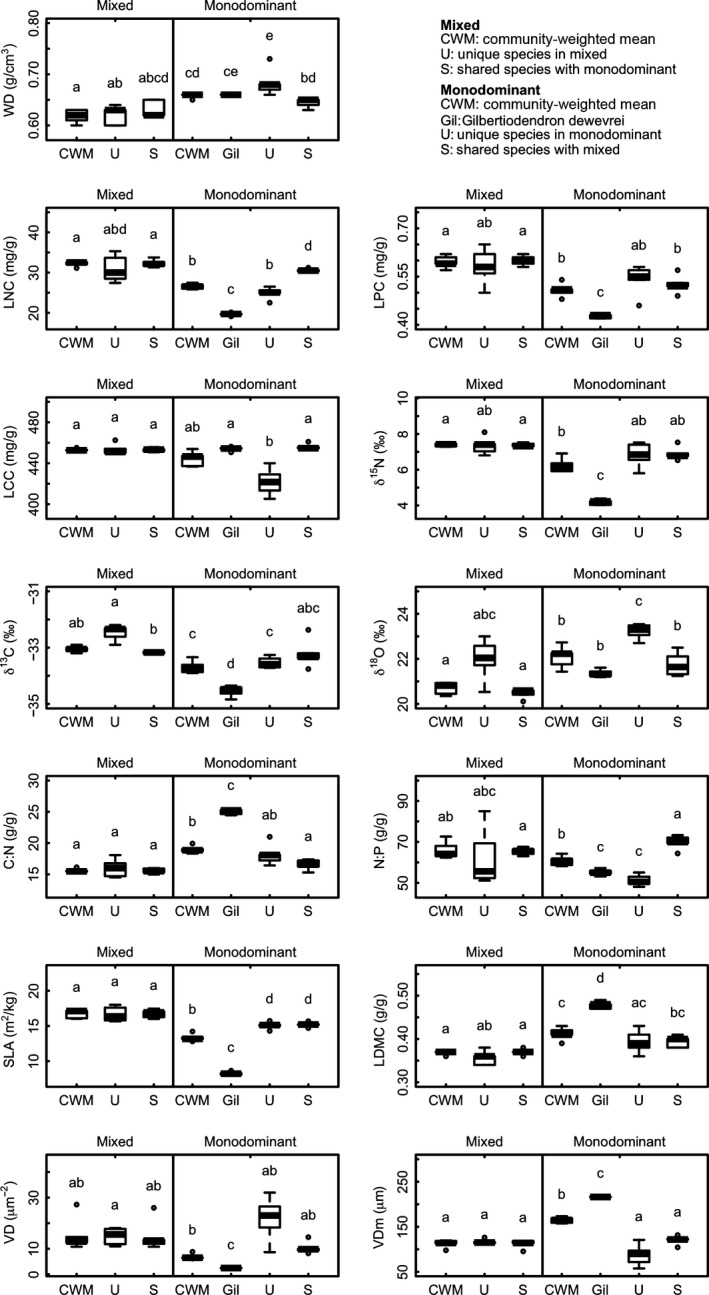
The mean of each individual trait is compared between species groups within the monodominant and the mixed forest. Within the monodominant forest, the dominant species *Gilbertiodendron dewevrei* (Gil) is compared to the trait space of all species unique for this forest (U for unique) and the species also present in the mixed forest (S for shared). Within the mixed forest, the species unique for the mixed forest (U) and the species also present in the monodominant forest are shown (S). For each forest type, the community mean is also indicated (CWM). Letters indicate whether there is a significant difference (*p* < .01) between all seven species groups. Trait abbreviations: wood density (WD), leaf nitrogen content (LNC), leaf phosphorus content (LPC), leaf carbon content (LCC), the isotopic composition of nitrogen (δ^15^N), carbon (δ^13^C) and oxygen (δ^18^O), CN ratio (C:N), NP ratio (N:P), specific leaf area (SLA), leaf dry matter content (LDMC), vessel density (VD), and vessel diameters (VDm)

Community‐weighted means (CWM) of traits within the monodominant forest are highly influenced by the dominance of *G. dewevrei* which makes up a large part of the community basal area, namely 65.3% (Table 1), although average *G. dewevrei* traits are generally different from the CWM (Figure 1). Moreover, a significant difference is found for most observed traits between *G. dewevrei* and other species in the community (Figure 1), indicating that *G. dewevrei* has a unique niche. The leaf nutrients of *G. dewevrei* are significantly lower for LNC (19.6 mg/g; compared to 27.7 mg/g for all other species in the community), δ^15^N (4.2‰; compared to 6.9‰), and LPC (0.43 mg/g; compared to 0.53 mg/g), and leaves have a higher C:N (25.0 g/g; compared to 17.3 g/g). Leaf investment traits also differ significantly with a lower SLA (8.3 m^2^/kg; compared to 15.1 m^2^/kg for all other species in the community) and a higher LDMC (0.48 g/g; compared to 0.39 g/g), although LCC (454 mg/g; compared to 438 mg/g) is similar to the other species. WD (0.66 g/cm^3^) is similar to other species in the community (0.66 g/cm^3^), but the VDm (216.3 μm; compared to 103.9 μm) is significantly higher and VD (2.4 per μm^2^; compared to 16.0 per μm^2^) significantly lower.

Significant differences have also been found between species within the monodominant tree community that are unique for the monodominant forest and species that are also present in the mixed forest. LNC of the unique species (24.9 mg/g) is significantly lower (*p* < .001) than that of the shared species (30.5 mg/g), although δ^15^N is similar (6.8‰–6.9‰). Additionally, the N:P ratio of the unique species (51.2 g/g) is significantly lower (*p* < .001) than that of the shared species (69.8 g/g) with values similar to *G. dewevrei* (55.1 g/g). The vascular wood traits also differ significantly (*p* < .05) between the unique and shared species in the monodominant forest, with unique species having higher VD (21.7 per μm^2^) and smaller VDm (87.3 μm) than the shared species (VD 10.4 per μm^2^; VDm 120.5 μm).

### 3.4 Monodominant vs mixed forest functional community

For most traits, a significant difference is found between CWM in mixed versus monodominant forests (Figure 1). Within the monodominant forest, we found lower nutrient contents (LPC 0.51 mg/g compared to 0.60 mg/g in the mixed forest, *p* < .001; LNC 26.6 mg/g compared to 32.2 mg/g, *p* < .001), lower δ^15^N (6.2‰; compared to 7.4‰, *p* < .001), thicker leaves (low SLA (13.3 m^2^/kg; compared to 16.7 m^2^/kg, *p* < .001), high LDMC (0.41 g/g; compared to 0.37 g/g, *p* < .001)), higher WD (0.66 g/cm^3^; compared to 0.62 g/cm^3^, *p* < .001) combined with lower VD (6.9 per μm^2^; compared to 15.6 per μm^2^, *p* < .05) and higher VDm (164.7 μm; compared to 112.5 μm, *p* < .001), and lower values for δ^13^C (−33.7‰; compared to −33.1‰, *p* < .001) combined with higher δ^18^O values (22.1‰; compared to 20.7‰, *p* < .001). These differences are not solely driven by the trait values of *G. dewevrei*. Namely, within the monodominant forest, unique and the shared species show significant shifts of mean trait values compared to the mixed forest (Figure 1). Leaf nitrogen content is significantly lower for unique and shared species in the monodominant forest (24.9 mg/g, *p* < .001; 30.5 mg/g, *p* < .01) and the C:N ratio is generally higher (unique 18.2 g/g, shared 16.5 g/g; *p* < .05), while LPC is lower for shared species in the monodominant forest compared to the mixed forest (0.53 mg/g, *p* < .001). Additionally, SLA remains lower in the monodominant tree community for all species groups (unique 15.0 m^2^/kg, *p* < .01; shared 15.2 m^2^/kg, *p* < .01). δ^18^O is higher for all species groups in the monodominant forest (unique 23.2‰, *p* < .001; shared 21.8‰, *p* < .01), while δ^13^C is only significantly lower (−33.5‰, *p* < .01) for the unique species compared to the mixed forest.

### 3.5 Mixed forest functional community

Species unique for the mixed forest—not present in the monodominant forest—only showed a difference δ^13^C and δ^18^O compared to species also present in the monodominant forest. Within the mixed forest, δ^13^C was higher (−32.5‰; compared to −33.2‰; *p* < .01) and δ^18^O was higher (22.0‰; compared to 20.5‰; *p* < .05). All other traits were similar between unique and shared species in the mixed forest.

## 4 Discussion

In this study, we investigate functional diversity and functional community structure of monodominant versus mixed tropical forest systems.

The monodominant and adjacent mixed forests differ significantly in terms of diversity. Firstly, lower species diversity in the tree community is found in the monodominant forest, confirming studies by Hart et al. (1989) and Peh (2009), although contradicting Makana et al. (2004). Secondly, lower functional richness is found in the monodominant forest, indicating a narrower functional niche space compared to the adjacent mixed forest (Mason et al., 2005; Villéger et al., 2008). Furthermore, the similarity in functional evenness and divergence between the two forest types shows that, even within the reduced niche space in the monodominant forests, a similar niche differentiation and trait distribution occur compared to the adjacent mixed forest (Paine, Baraloto, Chave, & Herault, 2011; Villéger et al., 2008). These indices thus show that the monodominant forest mainly differs from the adjacent mixed forest in the narrower range of its niche space, where a lower species diversity is present, which could be the result of environmental filtering (Mason et al., 2005; Villéger et al., 2008).

The monodominance by *G. dewevrei*, however, is a type of monodominance that is not clearly dependent on edaphic conditions with similar environmental conditions often being described for adjacent mixed forests (Conway, 1992; Hart, 1985; Peh, Sonké et al., 2011). Moreover, the establishment of this monodominance has been described by a series of possible, nonexclusive mechanisms and pathways (Peh, Lewis et al., 2011; Torti et al., 2001), irrespective of prevailing environmental conditions. In this study, indications are, however, found of local environmental conditions favorable for *G. dewevrei*.

First, monodominant *G. dewevrei* forests are often found to be sparsely distributed along rivers (Fayolle et al., 2014), which is also the case in our study area. Accordingly, *G. dewevrei* possesses an ensemble of traits related to water use and transport that could be favorable in this environment. Namely, its vascular traits with high VDm combined with low VD are not general for a late successional tropical species. These wide vessels have the advantage of a greater water transport capacity or hydraulic efficiency (Tyree & Zimmermann, 2002), but may also be more vulnerable to drought‐induced cavitation (Tyree & Sperry, 1989) although susceptibility to cavitation would need to be confirmed by pit membrane structure (Hacke & Sperry, 2001). Additionally, with δ^13^C being a proxy of the intrinsic water use efficiency (WUE; the ratio of photosynthetic carbon fixation to stomatal conductance; Dawson, Mambelli, Plamboeck, Templer, & Tu, 2002; Farquhar, Ehleringer, & Hubick, 1989) and δ^18^O providing a time‐integrated measure of stomatal conductance (Barbour, 2007; Farquhar, Cernusak, & Barnes, 2007; Hasselquist, Allen, & Santiago, 2010), simultaneous measurements of δ^13^C and δ^18^O indicate that *G. dewevrei* shows low WUE combined with a high stomatal conductance compared to other species in the community. This high stomatal conductance suggests little need for water loss regulation for *G. dewevrei* in this environment, and the large vessel size and low WUE indicate a limited drought resistance of *G. dewevrei*. This low drought resistance and small potential for water regulation possibly explain the presence of *G. dewevrei* near the rivers, where water tables are expected to be shallow. However, it should be noted that no information is available for our sites on the water status and year‐round averages and extremes in soil gravimetric water contents. Yet, *G. dewevrei*‐dominated forests do not always occur along rivers and forest streams. The traits described here for *G. dewevrei* reflect a known trade‐off with water transport capacity positively related to photosynthetic potential and carbon assimilation rates (Santiago et al., 2004) versus inhibited water conservation. As an upper canopy species, *G. dewevrei* might benefit more from increased potential carbon gain as opposed to safeguarding water conservation. In consequence, the distribution of *G. dewevrei* can be generally related to environments with sufficient water availability, and is not solely constrained to riverine locations.

Secondly, indications are found of environmental filtering through a reduction in N and P soil availability in the monodominant forest. We speculate that this difference with mixed forests is possibly caused by differences in mycorrhizal associations. Studies comparing monodominant and adjacent mixed forests often cannot identify differences in soil characteristics (Conway, 1992; Hart, 1985; Peh, Sonké et al., 2011; although see Torti et al., 2001). However, in our study area, lower bioavailable P concentrations were found in the 0–30 cm depth layer of the monodominant forest. Furthermore, we argue that based on leaf nutrient traits, two indications for N and P limitations can be found in our investigated forest systems. First, *G. dewevrei* was found to be significantly depleted in foliar δ^15^N indicating its association with ectomycorrhizal fungi (Craine et al., 2009; Hobbie & Högberg, 2012), confirming what has previously been reported for *G. dewevrei* (Onguene & Kuyper, 2001; Torti & Coley, 1999). Ectomycorrhizal fungi could affect the availability of inorganic N and possibly P present in soil (Corrales, Mangan, Turner, & Dalling, 2016). In our study, no difference was found in soil N between mixed and monodominant forest. However, only total N (including organic and inorganic N) has been measured (as is the case in Conway, 1992; Hart, 1985; Peh, Sonké et al., 2011) which might not represent the actual N available for plants. A fraction of total N could be sequestered in the ectomycorrhizal fungal biomass. In return, in the mixed forest, N and P limitations might be less as most tropical trees form arbuscular mycorrhizal associations (Corrales et al., 2016) that contribute to a smaller extent to N limitations (Smith & Read, 2008; although see Hodge & Fitter, 2010). Accordingly (and secondly), reduced leaf N and P contents are found at the community level for the monodominant forest, although these CWMs are highly influenced by *G. dewevrei* itself. Lower LNC values are, however, found for both the unique and shared species in the monodominant forest compared to the CWM of the mixed forest, as are the LPC values of the shared species in the monodominant forests. These reduced foliar N and P contents might indicate combined N and P limitations, possibly a direct influence of the immobilization of N and P by ectomycorrhizal fungi as discussed above. Furthermore, this alteration in N and P availability might be induced by the long‐term dominance of *G. dewevrei* itself, with slow‐decomposing leaf litter generated by the dominant *G. dewevrei* (i.e., low SLA, high LDMC, high C:N) reinforcing low nutrient turnover rates and low N and P availability (Brookshire & Thomas, 2013; Menge, 2011). Moreover, the presence of ectomycorrhizal fungi possibly suppresses decomposition rates even further due to competition with saprotrophic fungi (Fernandez & Kennedy, 2016). The alteration of the local environment resulting from the dominance of *G. dewevrei* has been suggested to be a monodominance‐enhancing mechanism (Peh, Lewis et al., 2011; Torti et al., 2001). *G. dewevrei* can namely thrive in this nutrient‐limited environment with its slow growth rates (deducted from its dense wood and thus high construction cost; Enquist, West, Charnov, & Brown, 1999) and high nutrient use efficiency (Peh, Lewis et al., 2011).

The environmental filtering encountered in the monodominant forest can also affect the subordinate species composition, namely by altering the probabilities of specific traits. For example, Peh et al. (2014) showed that species with low light requirement and high WD have a greater chance of establishing in the monodominant forest, where light levels are low under the dense canopy. Within our study area, this could not be confirmed with the WD of species uniquely found in the mixed forest being similar to those that did occur in the monodominant forest. However, overall community differences in traits—for the different species groups—have been found between the monodominant and the mixed forest, namely for nutrient contents, WUE traits, and SLA. The lower values of nutrient contents of all species within this monodominant tropical tree community could be a direct result of the altered N and P availability in this environment (Brookshire & Thomas, 2013; Peh, Lewis et al., 2011). Additionally, the species established in the monodominant forest show a lower mean SLA compared to the species in the mixed forest. These leaves with low SLA, high tissue density (see LDMC), and low nutrient concentrations (both N and P) generally have lower photosynthetic rates but a longer life span (Reich, Walters, Tjoelker, Vanderklein, & Bushena, 1998; Wright & Westoby, 2002). The longer leaf life span could provide advantages for species under the closed canopy of *G. dewevrei*, susceptible to the limited N availability, with an increased return of investment. Additionally, these leaf traits contribute to defend against herbivores and pathogens (Hanley, Lamont, Fairbanks, & Rafferty, 2007) and thus have higher survival rates (Poorter, Bongers, Sterck, & Wöll, 2003).

Water use efficiency ‐related traits of species not found in the monodominant forest provide an indication of the influence of an environmental filter related to the riverine locations. As discussed earlier, *G. dewevrei* showed a low WUE most likely caused by a high stomatal conductance, which is possibly beneficial in the moist soil conditions in these forests. We found that species in the mixed forest that have a high WUE (high δ^13^C) combined with a reduced stomatal conductance (high δ^18^O; Farquhar et al., 1989) did not establish in the monodominant forest. Species with a low WUE that keep their stomata open, thus reducing their stomatal resistance, could have a higher resource use efficiency for other limiting resources (e.g., light or nutrient limitations) enabling them to compete, or keep up, with the monodominant species. Further research is needed to confirm this hypothesis, because the isotopic compositions can only be used as proxies of WUE.

## 5 Conclusion

In conclusion, distinct differences in functional diversity and functional structure of the community were found between the monodominant and adjacent mixed forest. Hypothesis I, expressing that the *G. dewevrei* monodominant forests hold a lower functional diversity than the mixed forest, is confirmed, as is the prevalence of environmental filtering with indications found for two filters. First, the common location of *G. dewevrei* forests near forest rivers has been linked to its functional traits related to water use and transport. Secondly, collected trait data point toward a reduction in N and P availability in soils of the monodominant forest, possibly caused by ectomycorrhizal associations with *G. dewevrei*. Additionally, as proposed in Hypothesis II, a pattern between the trait ensemble of the monodominant *G. dewevrei* and the subordinate species that co‐occur with it in the same plots could be observed. This pattern was significantly different for species that only occur at the investigated mixed forest plots. More research will be necessary to distinguish the influence of the two encountered types of environmental filtering or their coregulation.

## Data Accessibility

All trait data will be contributed to the TRY database (www.try-db.org).

## Conflict of Interest

None declared.
