# Supplementary material for: Functional community structure of African monodominant Gilbertiodendron dewevrei forest influenced by local environmental filtering
Source: Ecol Evol. 2016 Dec 20;7(1):295–304. doi: 10.1002/ece3.2589 (PMC5216677; doi:10.1002/ece3.2589)
Supplement: Supplementary file 1 [file ECE3-7-295-s001.docx]

**Supplementary Information**


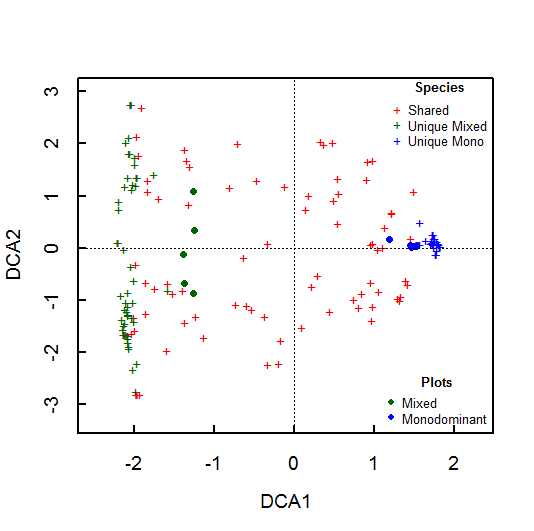


**Supplementary Figure S1:** Detrended correspondence analysis for 10 plots in mixed (n=5) and monodominant (n=5) forest, with species weighted by their basal area. Species in blue, green and red correspond to species group 1, 2 and 3 as defined in the main text.


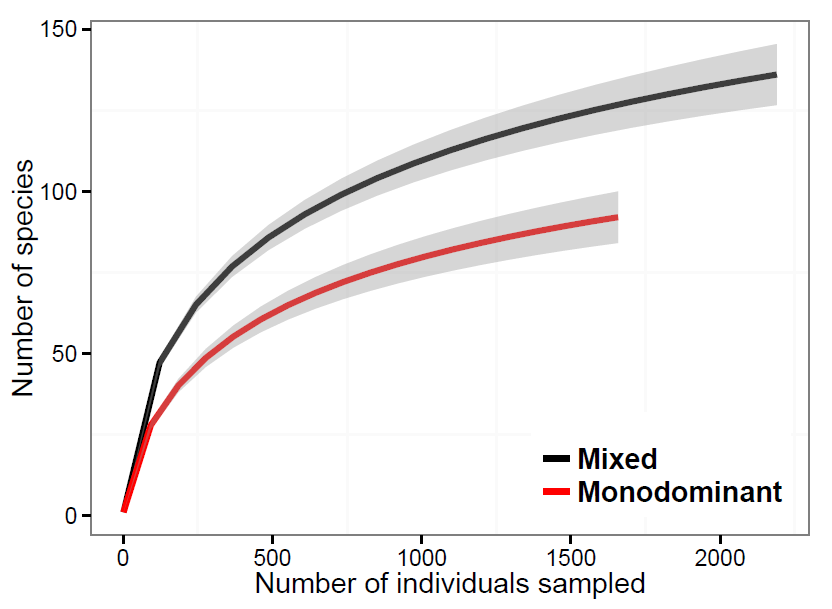


**Supplementary Figure S2:** Species richness of the mixed (black) and monodominant forest (red) presented by rarefaction curves. Standard errors are presented by grey backgrounds.
